# Anti-Inflammatory Activity of In Vitro Digested Manna in a Caco-2 and RAW264.7 Cells Co-Culture Model of Inflammatory Bowel Disease

**DOI:** 10.3390/antiox15050601

**Published:** 2026-05-09

**Authors:** Ilenia Concetta Giardina, Mussa Makran, Ignazio Restivo, Francesco Pappalardo, Guadalupe Garcia-Llatas, Maria Cristina Barbalace, Luisa Tesoriere, Antonio Cilla, Alessandro Attanzio

**Affiliations:** 1 Department of Biological, Chemical and Pharmaceutical Sciences and Technologies, University of Palermo, Via Archirafi 28, 90123 Palermo, Italy; ileniaconcetta.giardina@unipa.it (I.C.G.); ignazio.restivo@unipa.it (I.R.); luisa.tesoriere@unipa.it (L.T.); 2 Nutrition and Food Science Area, Faculty of Pharmacy and Food Sciences, University of Valencia, Av. Vicente Andrés Estellés s/n, 46100 Burjassot, Spain; mussa.makran@uv.es (M.M.); guadalupe.garcia@uv.es (G.G.-L.); 3 Department Analytics of R&D, Bionap s.r.l., C. da Fureria, Zona Industriale Ovest, Piano Tavola, 95032 Belpasso, Italy; francesco.pappalardo@bionap.com; 4 Department for Life Quality Studies, Alma Mater Studiorum, University of Bologna, Corso d’Augusto 237, 47921 Rimini, Italy; maria.barbalace2@unibo.it

**Keywords:** manna bioactivity, *Fraxinus* sp., anti-inflammatory, co-culture model, bioaccessible fraction, inflammatory bowel disease

## Abstract

Inflammatory bowel disease (IBD) involves intestinal barrier dysfunction and chronic inflammation. Manna, derived from the solidified phloem sap of *Fraxinus* species, is rich in mannitol and polyphenols and valued for its laxative, antioxidant, and anti-inflammatory properties. In this study, manna was digested in vitro to obtain its bioaccessible fraction (BFM), whose anti-inflammatory activity was tested in a Caco-2/RAW264.7 co-culture model. Caco-2 cells were pretreated with BFM (1/20 *v*/*v*, 6 mg/mL) 90 min before LPS stimulation (1 µg/mL, 24 h) of macrophages, using budesonide (1 μM) as reference. BFM pretreatment significantly reduced IL-8 secretion (70.8%) in Caco-2 cells, and IL-6 (43.1%) and TNF-α (83.1%) in RAW264.7 macrophages. It also improved redox balance in Caco-2 cells by decreasing iNOS (48.2%), NOx (33.2%), and ROS (26.4%), while stabilizing tight junctions through occludin upregulation (18.3%). Mechanistically, BFM downregulated NF-κB-COX-2-PGE_2_ signaling in macrophages, reducing NF-κB p65 nuclear translocation (65.6%), COX-2 levels (79.3%), and PGE_2_ production (50.8%). Co-treatment with budesonide showed antagonism for most markers (Combination Index (CI), 0.41–0.76), but additive/synergistic effects on ROS (CI, 1.06 ± 0.06) and NOx (CI, 1.10 ± 0.04). These findings highlight manna’s strong anti-inflammatory activity at a low, non-laxative dose (3.8 g/day), supporting its nutraceutical potential in IBD management.

## 1. Introduction

Inflammatory bowel disease (IBD) is mainly classified into Crohn’s disease (CD) and ulcerative colitis (UC). Although CD and UC share several phenotypic features, they can be distinguished by the site of inflammation within the gastrointestinal tract, their histological and immunological profiles, and the occurrence of disease-specific complications. CD is marked by transmural inflammation, which can affect any part of the gastrointestinal tract, whereas UC is limited to mucosal inflammation confined to the colon [1]. The development of these disorders involves multiple interacting factors, including alterations in gut microbiota composition, immune dysfunction, environmental influences, and genetic predisposition [2]. The evolving epidemiology of IBD suggests that environmental triggers—potentially linked to Westernized lifestyles, urbanization, dietary alterations, antibiotic use, and increased pollution—play a critical role in disease development [3]. Diet plays an important role in modulating both the development and progression of IBD by affecting the homeostasis of the intestinal barrier, microbiota, and immune system [4]. Western-style dietary patterns, typically high in saturated fats, processed meat, and refined sugars, alongside low fiber intake, represents a significant risk factor for IBD [5]. In contrast, adherence to the Mediterranean diet has been linked to a lower risk and reduced severity of IBD, largely due to its anti-inflammatory properties and positive impact on nutritional status [6,7]. For this reason, there is growing interest in functional foods rich in bioactive compounds as a promising strategy to address IBD, with the potential to prevent its manifestation and alleviate symptoms [8,9]. Manna is a natural product obtained from the solidification of phloem sap that exudes from incisions made in the trunks and branches of certain *Fraxinus* species (Oleaceae) during the summer. This sap condenses into “*manna in cannolo*”, which is relatively pure, and “*manna in rottame*” or “*manna in sorte*”, which are impure [10]. Sicilian manna is primarily obtained from *Fraxinus angustifolia* Vahl and *Fraxinus ornus* L., with production concentrated in the Madonie Mountains area in northern Sicily, where specific niche pedoclimatic conditions are found [10,11]. The manna used in this study was purified using a patented process (Patent No. 102015000061706), yielding a product with functional properties and high commercial value. Manna is a pharmacologically active product, renowned since ancient times for its beneficial properties as a gentle laxative without significant side effects, and has been traditionally used to treat various pathological conditions [11,12]. *Fraxinus* manna primarily contains D-mannitol, a compound known for its biological activities [12,13,14], as well as other mono- and oligosaccharides [15,16]. Recently, D-mannose has been linked to a reduction in colitis-related colorectal tumorigenesis by targeting tumor-associated macrophages in a DSS-treated murine model [17]. In addition, manna contains various bioactive phytochemicals recognized for their health-promoting properties [18,19,20,21,22,23], including elenolic acid, tyrosol, hydroxytyrosol (HT), catechin, fraxetin, verbascoside, gallic acid, procyanidin B1, and luteolin 3,7-glucoside [11,16]. Our recent studies demonstrated that the hydrophilic extract of manna (HME) from *Fraxinus angustifolia* Vahl exhibits strong reducing power and antioxidant properties in both chemical and biological in vitro assays. Additionally, HME prevented oxidative stress and inhibited the inflammatory response in differentiated Caco-2 cells stimulated by IL-1β [11]. It also showed anti-proliferative activity by inducing apoptosis via the mitochondrial pathway and causing cell cycle arrest in a human colon cancer cell line [24]. These findings suggest that the dietary intake of manna may represent a promising strategy for managing intestinal inflammation and offering chemopreventive effects, thereby prompting further investigation into its anti-inflammatory potential within the gastrointestinal tract. Recent studies have validated the anti-inflammatory effects of bioactive compounds and functional foods in preventing the onset of IBD using an in vitro Caco-2/RAW264.7 co-culture model of gut inflammation [25,26,27,28,29,30,31,32,33,34]. This co-culture system, with differentiated Caco-2 cells placed on the apical side of a Transwell plate and LPS-stimulated RAW264.7 macrophages on the basolateral side, simulates an in vivo inflammatory condition [25]. Furthermore, this configuration replicates the natural cellular organization of the intestinal mucosa, with epithelial cells forming the outer layer and immune cells located beneath. Through a permeable membrane, this intestinal barrier model also enables communication between different cell types, mimicking the intercellular interactions that occur in the pathophysiology of IBD [35]. The potential beneficial effects of functional foods on IBD primarily target the gastrointestinal tract. Consequently, assessing the activities of bioactive molecules derived from a food matrix after in vitro gastrointestinal digestion could provide more realistic conditions to evaluate the interactions occurring during intestinal absorption. In this study, we investigated, for the first time, the anti-inflammatory properties of the bioaccessible fraction of manna (BFM) against IBD using an in vitro co-culture model of Caco-2 intestinal cells and LPS-activated RAW264.7 macrophages. In addition, budesonide—a corticosteroid commonly used in the management of intestinal inflammation [36]—was employed as a reference compound for comparison and to evaluate potential pharmacological interactions with the BFM sample.

## 2. Materials and Methods

### 2.1. Reagents

2′,7′-dichlorofluorescein diacetate (DCFDA), 3-(4,5-dimethylthiazol-2-yl)-2,5-diphenyl-tetrazolium bromide (MTT), dimethyl sulfoxide (DMSO), dithiothreitol (DTT), sodium dodecyl sulfate (SDS), Dulbecco’s Modified Eagle’s Medium High Glucose (DMEM/HG), LPS from *Escherichia coli* O127, N-(1-naphthyl)ethylenediamine (NED), Tris-buffered saline (25 mM Tris, 150 mM NaCl, pH 7.4) (TBS), phosphatase inhibitor cocktail (100 mM sodium orthovanadate, 115 mM sodium molybdate, 400 mM sodium tartrate, and 200 mM imidazole), and protease inhibitor cocktail (50 mM benzenesulfonic fluoride, 15 mM aprotinin, 100 mM E-65, 50 mM EDTA, and 100 mM leupeptin) were purchased from Merck Lifescience S.R.L. (Madrid, Spain). Vanadium (III) chloride (VCl_3_) (>99%, *w*/*w*) was from Cymit Química (Barcelona, Spain). HPLC-grade solvents, including acetonitrile, methanol, water, and dimethylformamide, were purchased from Carlo Erba Reagenti (Milan, Italy). Analytical standards, including hydroxytyrosol, tyrosol, fraxetin, verbascoside, luteolin 3,7-glucoside, and catechin, were obtained from PhytoLab GmbH & Co. (Vestenbergsgreuth, Germany). When not specified, all chemicals and reagents were acquired from Sigma-Aldrich (Milan, Italy) and were of the highest available purity grade.

### 2.2. Samples

*Fraxinus angustifolia* Vahl is the common species of manna ash cultivated in the Madonie Natural Park, particularly concentrated in the Castelbuono and Pollina area (Palermo, Italy), and represents the primary botanical source of manna [10]. In August 2022, five distinct samples of low-grade, impure manna, derived from different *Fraxinus* trees, were harvested through bark scraping, pooled, and purified to remove contaminants using the patented procedure developed at the University of Palermo (Patent no. 102015000061706). The five collected batches were unified and analyzed as a single composite sample, since manna does not come from a single tree but rather from a mix of trees. The pure ivory-white manna powder obtained was then stored in a dry, dark place at room temperature for one month prior to undergoing in vitro gastrointestinal digestion.

### 2.3. INFOGEST 2.0 In Vitro Gastrointestinal Digestion

In vitro gastrointestinal digestion of purified manna was carried out according to the standardized INFOGEST 2.0 protocol [37] to isolate the BFM, which was subsequently employed to assess its biological effects on in vitro co-culture model. Specifically, 5 g of manna was mixed with 3.5 mL of simulated salivary fluid, 0.5 mL of α-amylase solution (final concentration of 75 U/mL), 25 μL of 0.3 M calcium chloride, and 975 μL of ultrapure water, culminating in a final volume of 10 mL. This oral bolus was then incubated in a shaking water bath for 2 min at 37 °C and 95 rpm. Following the oral digestion phase, 7.5 mL of simulated gastric fluid, 0.98 mL of rabbit gastric extract (resulting in a final lipase concentration of 60 U/mL), 0.62 mL of pepsin solution (final concentration of 2000 U/mL), and 5 μL of 0.3 M calcium chloride were incorporated, with manual mixing for 1 min. The pH was subsequently adjusted to 3, and ultrapure water was added to bring the final volume to 20 mL. The resultant gastric mixture was incubated in a shaking bath at 37 °C and 95 rpm for 2 h. For the intestinal phase, 11 mL of simulated intestinal fluid, 5 mL of pancreatin solution (final concentration of 100 U/mL), 0.1 mL of cholesterol esterase solution (final activity of 0.075 U/mL), 40 μL of 0.3 M calcium chloride, and 2.5 mL of bovine bile solution (final concentration of 10 mM) were added to the gastric digesta. The mixture was manually stirred for 1 min, the pH adjusted to 7, and ultrapure water added to achieve a final volume of 40 mL. This intestinal mixture underwent incubation in a shaking bath for an additional 2 h at 37 °C and 95 rpm. The final digesta was then centrifuged (90 min, 4 °C, 3100× *g*) to collect the supernatant, which represented the BFM. All digestion experiments were performed in triplicate, and the obtained BFM was sterilized through filtration with a 0.22 μm filter. To validate that digestion reagents did not exert adverse effects on cell cultures and to ensure compatibility for subsequent cell assays [38], a blank of digestion (BD) was obtained by submitting 5 g of ultrapure water to the digestion process.

### 2.4. Chromatographic Analysis of Phenolic Compounds

To evaluate the bioaccessible phenolic profile of Manna after gastrointestinal digestion, both the undigested manna and its bioaccessible fraction obtained through in vitro digestion were analyzed. Additionally, BD was included to identify and exclude any phenolic compounds that could originate from the digestion reagents themselves. Phenolic compounds were analyzed using a previously validated chromatographic method [11]. Aliquots of Manna, BFM, and BD were purified using Bond Elut C18 solid-phase extraction cartridges (500 mg, 6 mL; Agilent Technologies, Santa Clara, CA, USA) according to the manufacturer’s instructions. The eluates were evaporated under a stream of nitrogen (Rapid Mini, Crescent Scientific, Mumbai, India) and reconstituted in dimethylformamide/water (9:1, *v*/*v*) to a final concentration of 30 mg manna equivalent/mL. HPLC analyses were conducted in duplicate using an Agilent 1100 Infinity system equipped with a diode array detector (DAD) and a 150 × 4.6 mm i.d., 2.7 μm Ascentis Express C18 column. The chromatographic separation was performed using a solvent system consisting of H_2_O/H_3_PO_4_ (99:1, solvent A) and methanol/acetonitrile/H_3_PO_4_ (49.5:49.5:1, solvent B). The gradient program was as follows: 95% A to 77% A over 34 min, maintained at 77% A for 3 min, 74% A at 60 min, 60% A at 85 min, 20% A at 90 min, and 0% A at 92 min, for a total run time of 105 min. Column temperature was 25 °C, flow rate 1 mL/min, and injection volume 15 μL. Chromatograms were recorded from 190 to 500 nm and monitored primarily at 280 ± 2 nm. The comparison between the undigested Manna and the BFM allowed determination of bioaccessibility, expressed as the percentage of each compound remaining after in vitro digestion relative to its content in the original extract.

### 2.5. Determination of Total Polyphenol Content

To assess the impact of in vitro digestion on polyphenol availability, the total polyphenol content (TPC) of manna was quantified before and after digestion using the Folin–Ciocalteu assay [39]. Briefly, a 100 µL aliquot (either BFM or a water suspension of manna at 100 mg/mL, this concentration being selected as it provided the best dissolution) was mixed with 3 mL of 2% (*w*/*v*) sodium carbonate solution and 100 µL of 1 N Folin–Ciocalteu reagent. The mixture was incubated at room temperature in the dark for 1 h, after which the absorbance was measured at 765 nm using a UV–VIS spectrophotometer (Lambda 365, Perkin-Elmer, Waltham, MA, USA). A calibration curve was constructed using gallic acid (10–300 mg/L), and TPC values were calculated by interpolation from the calibration curve (y = 0.0014x; R^2^ = 0.9854). Results were expressed as mg of gallic acid equivalents (GAE) per 100 g of manna.

### 2.6. Cell Cultures

Human colonic epithelial adenocarcinoma (Caco-2) cells and murine macrophage/monocyte (RAW264.7) cell lines were purchased from the American Type Culture Collection (ATCC, Rockville, MD, USA). Both cell lines, used between passages 10 and 20, were cultured in Dulbecco’s Modified Eagle’s Medium High Glucose (4.5 g/L glucose) (DMEM/HG), supplemented with 10% (*v*/*v*) fetal bovine serum (FBS), 100 U/mL penicillin, and 100 µg/mL streptomycin. Cells were maintained at a density of 5.0 × 10^4^ cells/cm^2^ in 75 cm^2^ flasks under controlled conditions at 37 °C, with 5% (*v*/*v*) CO_2_ and 95% relative humidity. Prior to conducting the experimental assays, Caco-2 cell differentiation was confirmed to ensure enterocyte-like characteristics. Cells were seeded onto Transwell^®^ inserts at a density of 80,000 cells/cm^2^ and maintained under standard culture conditions. Differentiation was assessed daily by two complementary approaches: measurement of transepithelial electrical resistance (TEER) using a Millicell ERS 3.0 digital voltohmmeter (Merck Millipore, Billerica, MA, USA), with values ≥ 800 Ω·cm^2^ indicating the establishment of tight junctions and functional monolayer integrity [35]; and microscopic evaluation of dome formation, recognized as a characteristic morphological marker of polarized, functionally differentiated Caco-2 cells.

### 2.7. Cell Viability Assay

To preliminarily assess the optimal incubation time and dilution of BFM that does not affect cell viability in differentiated Caco-2 cells, to investigate its anti-inflammatory effects, cell viability was assessed using the MTT colorimetric assay, as previously reported [34]. For this purpose, Caco-2 cells were plated at a density of 26,500 cells per well in a 96-well plate, each containing 200 µL of DMEM/HG, and incubated for 8 days with medium replacement every 2 days to induce differentiation into normal intestinal-like cells [40]. After differentiation, cells were exposed to BD and BFM, diluted in fresh culture medium at ratios of 1/10, 1/20, and 1/30 (*v*/*v*), and incubated for different time points (30, 60, and 90 min). Subsequently, the medium supplemented with BD and BFM was discarded, the cells were washed and then incubated with fresh medium containing MTT reagent (0.1 mg/mL) at 37 °C for 2 h. After removing the MTT solution, the precipitated formazan crystals were dissolved in DMSO, and the absorbance of the resulting chromogenic solution was measured using a multi-well spectrophotometer at 570 nm (LTEK A-302 plate reader, INNO, Seongnam, Republic of Korea). The absorbance value of untreated cells (control) was considered as 100% viability [24].

### 2.8. Co-Culture Model of Intestinal Inflammation

The co-culture model of differentiated Caco-2 cells and RAW264.7 macrophages was developed based on the protocol by Tanoue et al. [25], with modifications of the LPS concentration and exposure time (1 μg/mL for 24 h) to achieve effective stimulation of the cellular system [34,35]. Briefly, Caco-2 cells were seeded at a density of 9 × 10^4^ cells per well in the apical chamber of Transwell^®^ 12-well plates (membrane area: 1.12 cm^2^; pore size: 0.4 µm; Corning CoStar Corp., Cambridge, MA, USA). One day prior to initiating the assays with 8-day differentiated Caco-2 cells, RAW264.7 macrophages were plated at a density of 3.4 × 10^5^ cells per well in the basolateral compartment of the Transwell^®^ plates and cultured for 24 h. After the differentiation period, Transwell^®^ inserts containing Caco-2 cell monolayers were transferred into 12-well plates, pre-seeded with RAW264.7 cells in the basolateral side. Appropriately diluted samples of BFM and BD (1/20, *v*/*v*) were then added into the apical compartment and incubated for 90 min. Budesonide was employed as a reference compound due to its well-established glucocorticoid activity and clinical use as an anti-inflammatory drug in intestinal disorders. To compare the effects and evaluate potential pharmacological interactions between the BFM and budesonide, the drug was added into the apical side at a concentration of 1 μM [34,35], either alone or in combination with BFM. This approach allowed us to determine not only the relative efficacy of BFM compared to a pharmacological standard but also whether the presence of both agents produced additive, synergistic, or antagonistic effects on the modulation of inflammatory responses in the co-culture model. Following 90 min of incubation, treatments were replaced with the addition of fresh culture medium, and macrophages were stimulated by LPS at a concentration of 1 μg/mL for 24 h. Cells not treated with BFM or not exposed to LPS were used as a negative control.

### 2.9. Assessment of Pro-Inflammatory Mediators Release

The concentration of inflammatory mediators secreted by differentiated Caco-2 cells (IL-8) in the apical medium and by RAW264.7 macrophages (IL-6, TNF-α, and PGE_2_) in the basolateral medium was quantified using ELISA kits, following the manufacturer’s instructions (Invitrogen, Frederick, MD, USA).

### 2.10. Measurement of Reactive Oxygen Species

Reactive oxygen species (ROS) production was quantified in differentiated Caco-2 cell monolayers using flow cytometry, measuring fluorescence changes induced by cytosolic oxidation of the DCFDA probe [11]. Briefly, DCFDA was added to the culture medium at a final concentration of 10 μM, 30 min before the end of the cell treatment. After incubation with the probe, the culture medium was discarded, and the cells were washed with phosphate-buffered saline (PBS), resuspended in the same buffer, and immediately subjected to flow cytometric analysis (CytoFLEX System B2-R2-V0 Beckman Coulter, Brea, CA, USA). The fluorescence intensity of at least 10^4^ events per sample was measured in the FL-1 channel, with excitation at 488 nm and emission at 530 nm.

### 2.11. Nitrite/Nitrate Determination

The quantification of NO production by differentiated Caco-2 cells in the apical medium was performed by measuring total nitrite and nitrate (NOx) concentrations using the VCl_3_/Griess assay, as described in a previously established protocol [41]. In brief, 100 μL of the apical culture medium was combined with 100 μL of 0.8% (*w*/*v*) VCl_3_ (prepared in 1 M HCl), 50 μL of 2% (*w*/*v*) sulfanilamide (in 5% (*v*/*v*) H_3_PO_4_), and 50 μL of 0.1% (*w*/*v*) NED (in ultrapure water) in a 96-well microplate. The mixture was incubated in the dark at 37 °C for 30 min. Following incubation, absorbance was measured at 540 nm using a spectrophotometric plate reader. NOx concentrations were determined by interpolating the absorbance values against an external calibration curve generated using sodium nitrate standards, with concentrations ranging from 1.56 to 100 μM.

### 2.12. Western Blot Analysis

Protein levels were quantified by Western blotting, following the methodology described by Restivo et al. [42]. For protein extraction from RAW264.7 macrophages, cells were lysed in a hypotonic buffer containing 10 mM HEPES, 1.5 mM MgCl_2_, 10 mM KCl, 0.1 mM DTT, 1% (*v*/*v*) phosphatase inhibitor, and 1% (*v*/*v*) protease inhibitor, and incubated on ice for 15 min. The lysates were then centrifuged at 12,000× *g* for 20 min at 4 °C to isolate the cytosolic fraction (supernatant). The resulting cell pellets were resuspended in lysis buffer containing 20 mM HEPES, 25% glycerol, 0.42 mM NaCl, 1.5 mM MgCl_2_, 0.2 mM EDTA, 0.1 mM DTT, 1% (*v*/*v*) phosphatase inhibitor, and 1% (*v*/*v*) protease inhibitor and incubated on ice for 30 min. The samples were then centrifuged at 20,000× *g* for 10 min at 4 °C to obtain the nuclear fraction. For differentiated Caco-2 cells, protein extraction was conducted using ice-cold RIPA lysis buffer composed of 50 mM Tris-HCl (pH 7.4), 150 mM NaCl, 1% Triton X-100, 0.1% SDS, 1% (*v*/*v*) phosphatase inhibitor, and 1% (*v/v*) protease inhibitor. Lysates were centrifuged at 12,000× *g* for 10 min at 4 °C, and the recovered supernatants represented whole-cell protein extract. Protein concentration was determined by Bradford assay. For each sample, equal amounts of protein (50 µg per lane) were separated using discontinuous 10% SDS-PAGE and subsequently transferred onto a PVDF membrane (Millipore, Cat. No. IPVH00010) via electroblotting. Membranes were incubated in a blocking solution containing 5% non-fat dry milk solution for 1 h at room temperature, and subsequently incubated overnight at 4 °C with primary antibodies (Santa Cruz Biotechnology, Inc., Dallas, TX, USA) prepared in Tris-buffered saline with 1% (*v/v*) Tween 20 (TBST) and 5% (*w/v*) BSA. Mouse monoclonal anti-iNOS (sc-7271), anti-occludin (sc-133256), anti-NF-κB p65 (sc-372) and anti-Cox-2 (sc-1745) primary antibodies were used at a dilution of 1/500 (*v*/*v*). Following three washes with TBST, the membranes were incubated for 2 h at room temperature with HRP-conjugated secondary antibodies (Sigma-Aldrich, Cat. No. AP160P) at a dilution of 1/2000 (*v/v*). After five additional washes with TBST, the immunoblots were developed using the Enhanced Chemiluminescent Substrate (ECL™ Prime Western Blotting System, Marlborough, MA, USA). Mouse monoclonal anti-β-Actin (sc-47778) and anti-Lamin B1 (sc-374015) primary antibodies were used as reference proteins for densitometric normalization and as loading controls. The chemiluminescent signal intensity of the protein blots was quantified using a C-Digit Blot Scanner (LI-COR, Lincoln, NE, USA), and the results were expressed as arbitrary densitometric units, analyzed with ImageJ software (version 1.54d). Western blot membranes corresponding to the representative experiments used for densitometric analysis are provided in Supplementary Materials (Figure S2).

### 2.13. Estimation of Combination Index

Combination index (CI) was calculated based on the methodology described by Chou (1984) [43] and adapted by Álvarez-Sala et al. [44] to assess the interaction between BFM and budesonide in reducing pro-inflammatory mediator levels. The CI was determined using the formula: CI = (% BFM + budesonide)/(% BFM + % budesonide), where “% BFM” and “% budesonide” represent, respectively, the percentage of reduction achieved by BFM and budesonide individually, and “% BFM + budesonide” represents the percentage of reduction achieved by the combined treatment. Values of CI < 1 indicate antagonism, CI > 1 than 1 indicate synergism, and a CI = 1 represents an additive effect.

### 2.14. Statistical Analysis

Results are expressed as mean ± standard deviation (SD). Unless otherwise stated, each experiment was conducted independently three times (biological replicates), with a single measurement per experiment (technical replicate). Data analysis and graphical representations were carried out using GraphPad InStat version 3.0 statistical software (GraphPad Software, Inc., San Diego, CA, USA), applying a normality test followed by ANOVA with Tukey’s post hoc correction for multiple comparisons. Statistical significance was determined at *p* < 0.05.

## 3. Results

### 3.1. Phenolic Profile and Total Phenolics in Manna and Its Bioaccessible Fraction

The HPLC-DAD analysis revealed that several phenolic compounds were detectable both in the undigested Manna extract and in the BFM obtained after in vitro gastrointestinal digestion. Table 1 summarizes the concentration of each phenolic compound before and after digestion, as well as the calculated bioaccessibility (%). Representative HPLC-DAD chromatograms of undigested Manna and the BFM are provided in Supplementary Materials (Figure S1).

HT, tyrosol, catechin, luteolin 3,7-glucoside, and verbascoside were detected in both the undigested Manna extract and the BFM, indicating their potential availability for intestinal absorption. Fraxetin was below the limit of quantification in both samples. The total phenolic content decreased from 33.85 ± 1.09 mg/kg in the undigested extract to 13.47 ± 0.68 mg/kg in the BFM, corresponding to an overall bioaccessibility of 39.8 ± 0.88%. HT and verbascoside exhibited the highest relative bioaccessibility (48.5% and 41.6%, respectively), while catechin, luteolin 3,7-glucoside, and tyrosol showed lower retention after digestion (31.9%, 36.0%, and 36.1%, respectively). Although HT and verbascoside are the most bioaccessible phenolics, the compounds present at the highest absolute concentrations in the BFM are HT and tyrosol, which likely contribute most to the biological activity of the fraction.

After the characterization of the phenolic profile, the TPC of manna and its bioaccesible fraction was determined to have a general overview on the impact of gastrointestinal digestion on the main antioxidants of the extracts. In this sense, a marked decrease in TPC was observed after digestion. The initial TPC was 198 ± 10 mg GAE/100 g, which dropped to 7.65 ± 0.98 mg GAE/100 g in the bioaccessible fraction, corresponding to a bioaccessibility of 3.9 ± 0.5%.

### 3.2. Effects of BFM on Caco-2 Cell Viability

Before investigating the anti-inflammatory effects in the co-culture cell model, the effects of BFM and BD on the viability of differentiated Caco-2 cells were assessed to determine the optimal treatment conditions. As shown in Table 2, BD did not reduce the viability of Caco-2 cells, regardless of the tested dilutions (1/10, 1/20, and 1/30, *v*/*v*) or exposure times (30, 60, and 90 min). At the lowest dilution (1/10, *v*/*v*), BFM significantly reduced cell viability across all three exposure intervals, with decreases of 26.1%, 26.3%, and 27.3%, respectively, compared to untreated cells (*p* < 0.05). In contrast, the 1/20 and 1/30 (*v/v*) dilutions did not affect cell viability. Considering the results obtained, subsequent assays to evaluate the anti-inflammatory activity of BFM were performed using the 1/20 (*v/v*) dilution, pre-incubated for 90 min. This treatment condition enables the use of the highest concentration of bioactive phytochemicals contained in the manna for the maximum possible duration, without affecting cell viability.

### 3.3. BFM Modulates Inflammation by Reducing Cytokine Release

**Figure 1:**
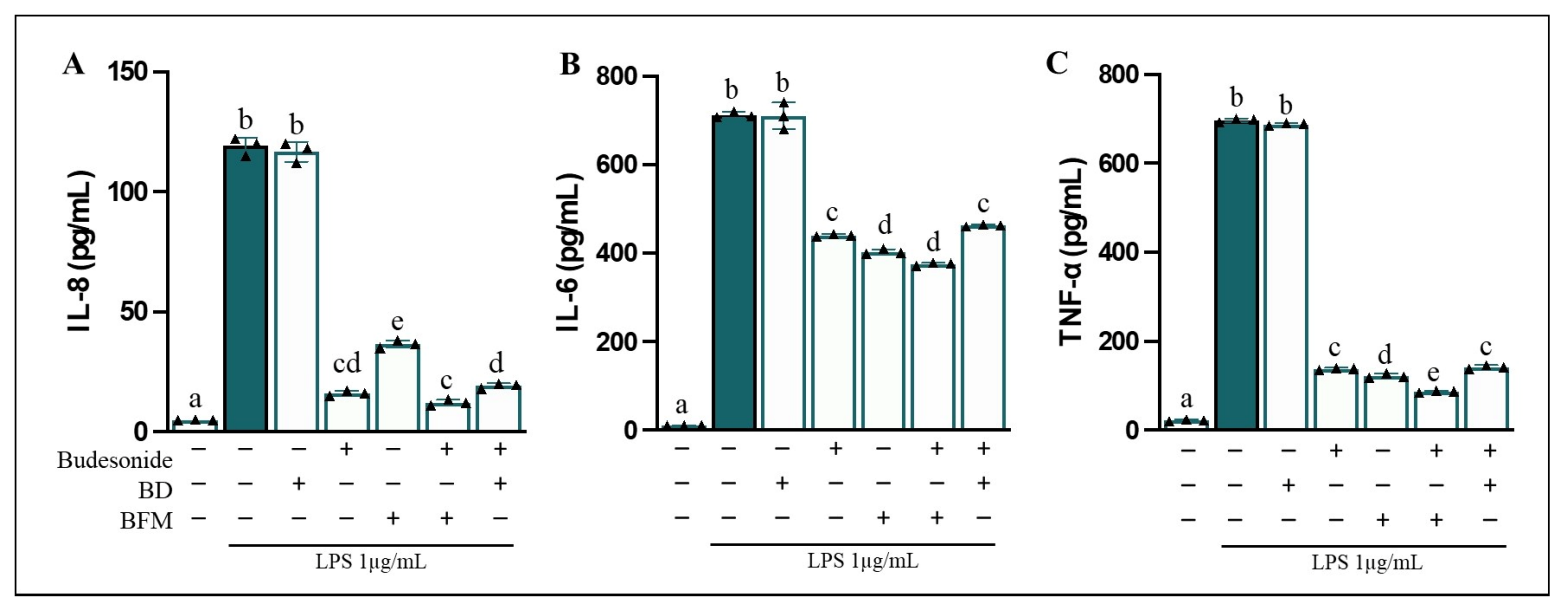
**Measurement of pro-inflammatory cytokine secretion levels in the co-culture system.** Analyses were conducted evaluating (**A**) IL-8 levels in the apical medium (differentiated Caco-2 cells) and (**B**) IL-6 and (**C**) TNF-α levels in the basolateral medium (RAW264.7 cells). Caco-2 cells were pre-treated for 90 min with BD, BFM (1/20, *v*/*v*), and budesonide (1 µM, positive control), as indicated by (+), while absence of treatment is indicated by (−). Subsequently, RAW264.7 macrophages were stimulated with LPS at a concentration of 1 µg/mL for 24 h. Data are expressed as mean ± SD of three experiments conducted in triplicate. Different letters (a–e) indicate statistically significant differences among all groups within each panel (*p* < 0.05) (one-way ANOVA followed by Tukey’s test).

Pro-inflammatory cytokines are key mediators involved in the regulation of inflammatory responses, acting as key immune mediators and important biomarkers for assessing the activation and progression of inflammatory processes [45]. The anti-inflammatory activity of BFM was initially evaluated by analyzing its effects on the quantification of pro-inflammatory cytokine secretion in a Caco-2/RAW264.7 co-culture system following stimulation (Figure 1). In this regard, compared to the untreated control, differentiated Caco-2 cells exhibited a substantial increase (*p* < 0.05) in IL-8 release (Figure 1A) into the apical medium, with a 24.3-fold change following stimulation of RAW264.7 macrophages in the basolateral compartment with LPS (1 µg/mL) for 24 h. Simultaneously, LPS-treated RAW264.7 cells showed a significant increase (*p* < 0.05) in IL-6 (Figure 1B) and TNF-α secretion (Figure 1C) into the basolateral medium, with fold change of 66.9 and 28.4, respectively, compared to control cells. In contrast, a 90-min pre-treatment with BD at a 1/20 (*v*/*v*) dilution did not affect the release of LPS-induced pro-inflammatory cytokines in either cell line compared to cells exposed to LPS alone. However, under the same experimental conditions, pre-incubation with BFM demonstrated protective effects by significantly attenuating (*p* < 0.05) cytokine release, reducing IL-8 levels by 70.8%, IL-6 by 43.1%, and TNF-α by 83.1% relative to LPS-induced stimulation. The decreases in IL-8, IL-6, and TNF-α levels observed with BFM were comparable to those achieved following pre-treatment with 1 µM budesonide (86.5%, 38.5%, and 80.1%, respectively), underscoring its strong efficacy in contrasting intestinal inflammation. Additionally, co-incubation of BFM with budesonide exhibited effects similar to those observed with the respective individual treatments. Since BD lacked significant effects, it was excluded from the analysis of following experimental parameters.

### 3.4. BFM Enhances Cellular Oxidative/Nitrosative Balance

**Figure 2:**
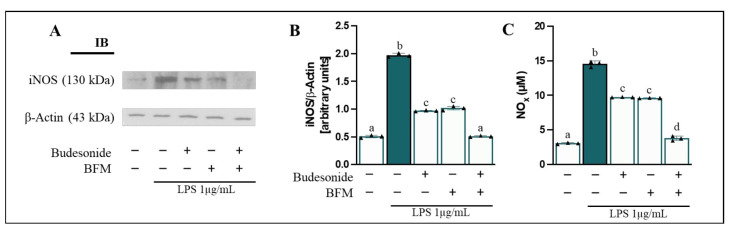
**Evaluation of iNOS protein levels in differentiated Caco-2 cells and NOx release into the apical medium.** Caco-2 cells were pre-treated for 90 min with BFM (1/20, *v*/*v*) and budesonide (1 µM, positive control), as indicated by (+), while absence of treatment is indicated by (−). Subsequently, RAW264.7 macrophages were stimulated with LPS at a concentration of 1 µg/mL for 24 h. (**A**) Representative immunoblot (IB) images and (**B**) densitometric analysis of iNOS levels normalized to β-Actin. (**C**) NOx concentration. Data are expressed as mean ± SD (*n* = 3). Different letters (a–d) indicate statistically significant differences among all groups within each panel (*p* < 0.05) (one-way ANOVA followed by Tukey’s test).

Excessive ROS and iNOS-derived NO imbalance redox homeostasis, promote oxidative stress, epithelial damage, and chronic inflammation in IBD [2]. Therefore, iNOS protein levels in differentiated Caco-2 cells, NOx release into the apical medium, and intracellular ROS production were assessed. Our findings, presented in Figure 2, demonstrated that LPS stimulation in the basolateral medium resulted in an increase (*p* < 0.05) in iNOS protein levels (3.9-fold change) (Figure 2A,B) and concurrent rise in NOx concentration (4.7-fold change) (Figure 2C) compared to control cells.

**Figure 3:**
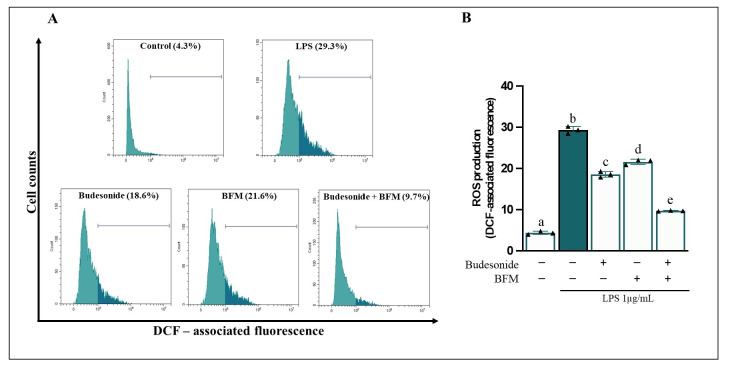
**Measurement of intracellular ROS production in differentiated Caco-2 cells.** Caco-2 cells were pre-treated for 90 min with BFM (1/20, *v*/*v*) and budesonide (1 µM, positive control), as indicated by (+), while absence of treatment is indicated by (−). Subsequently, RAW264.7 macrophages were stimulated with LPS at a concentration of 1 µg/mL for 24 h. (**A**) Representative fluorescence histograms and (**B**) quantitative analysis of ROS generation. Data are expressed as mean ± SD of three experiments conducted in triplicate. Different letters (a–e) indicate statistically significant differences among all groups within each panel (*p* < 0.05) (one-way ANOVA followed by Tukey’s test).

Furthermore, LPS exposure led to an elevation in intracellular ROS generation (6.8-fold change) (Figure 3). Pre-incubation of Caco-2 cells with BFM significantly reduced (*p* < 0.05) iNOS protein levels by 48.2% and NOx release by 33.2% compared to the LPS-treated condition. Additionally, BFM decreased intracellular ROS levels by 26.4%. In the same manner, pre-treatment with budesonide significantly attenuated (*p* < 0.05) iNOS protein levels by 50.7%, extracellular NOx release by 34.1%, and intracellular ROS production by 36.8%. Combination of BFM with budesonide demonstrated enhanced activity compared to either treatment alone.

### 3.5. BFM Maintains Intestinal Epithelial Barrier Integrity

**Figure 4:**
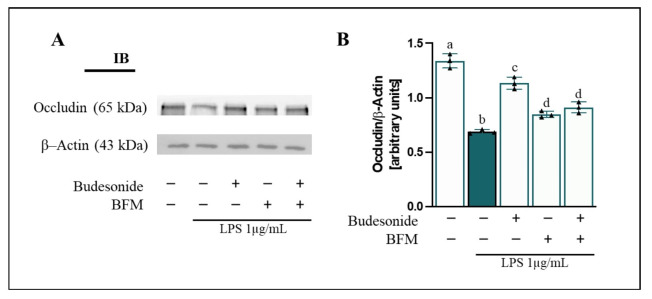
**Assessment of occludin protein levels in differentiated Caco-2 cells.** Caco-2 cells were pre-treated for 90 min with BFM (1/20, *v*/*v*) and budesonide (1 µM, positive control), as indicated by (+), while absence of treatment is indicated by (−). Subsequently, RAW264.7 macrophages were stimulated with LPS at a concentration of 1 µg/mL for 24 h. (**A**) Representative immunoblot (IB) images and (**B**) densitometric analysis of occludin levels normalized to β-Actin. Data are expressed as mean ± SD (*n* = 3). Different letters (a–d) indicate statistically significant differences among all groups within each panel (*p* < 0.05) (one-way ANOVA followed by Tukey’s test).

Occludin regulates tight junctions (TJ) in the intestinal epithelium, maintaining barrier integrity, and its dysfunction is associated with the pathogenesis of IBD, promoting increased intestinal permeability and immune system dysregulation [46,47]. In this context, occludin protein levels in intestinal epithelial monolayers were measured. As shown in Figure 4, addition of LPS into the basolateral compartment caused a reduction (*p* < 0.05) in occludin protein levels (2.05-fold change) in differentiated Caco-2 cells compared to control cells. Pre-treatment with BFM and budesonide improved (*p* < 0.05) occludin levels in Caco-2 cells by 18.3% and 43.7%, respectively, compared to LPS-exposed cells. In addition, BFM tested alone produced results comparable to those obtained with co-incubation of budesonide.

### 3.6. BFM Downregulates Inflammation-Related Pathway

**Figure 5:**
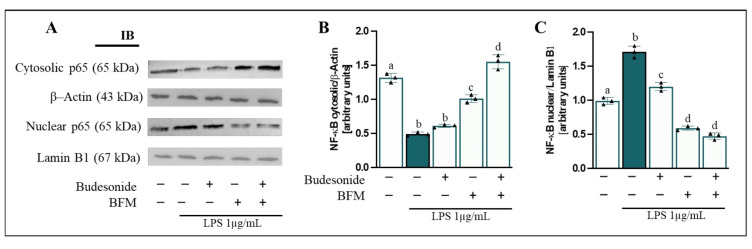
**Evaluation of NF-κB p65 nuclear translocation levels in RAW264.7 cells.** Caco-2 cells were pre-treated for 90 min with BFM (1/20, *v*/*v*) and budesonide (1 µM, positive control), as indicated by (+), while absence of treatment is indicated by (−). Subsequently, RAW264.7 macrophages were stimulated with LPS at a concentration of 1 µg/mL for 24 h. (**A**) Representative immunoblot (IB) images and (**B**,**C**) densitometric analysis of NF-κB p65 levels in cytosolic and nuclear fractions, normalized to β-Actin and Lamin B1, respectively. Data are expressed as mean ± SD (*n* = 3). Different letters (a–d) indicate statistically significant differences among all groups within each panel (*p* < 0.05) (one-way ANOVA followed by Tukey’s test).

The NF-κB-COX-2-PGE_2_ signaling axis plays a key role in regulating chronic inflammation in IBD, modulating immune cell activation and pro-inflammatory cytokine production [48]. In this regard, NF-κB activation, indicated by nuclear translocation of the p65 subunit, as well as COX-2 protein levels and its bioactive lipid product PGE_2_, were investigated in RAW264.7 macrophages. LPS-stimulated RAW264.7 cells exhibited a significant increase (*p* < 0.05) in nuclear translocation of NF-κB p65 (1.7-fold change) and a concomitant decrease in cytosolic levels (62.8%) compared to control cells (Figure 5).

**Figure 6:**
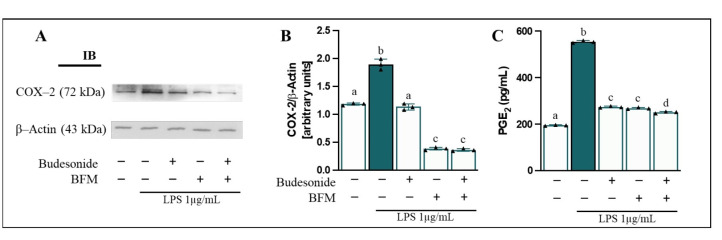
**Assessment of COX-2 protein levels in RAW264.7 cells and PGE_2_ secretion into the basolateral medium.** Caco-2 cells were pre-treated for 90 min with BFM (1/20, *v*/*v*) and budesonide (1 µM, positive control), as indicated by (+), while absence of treatment is indicated by (−). Subsequently, RAW264.7 macrophages were stimulated with LPS at a concentration of 1 µg/mL for 24 h. (**A**) Representatives immunoblot (IB) images and (**B**) densitometric analysis of COX-2 levels normalized to β-Actin. (**C**) PGE_2_ concentration. Data are expressed as mean ± SD (*n* = 3). Different letters (a–d) indicate statistically significant differences among all groups within each panel (*p* < 0.05) (one-way ANOVA followed by Tukey’s test).

Furthermore, an enhancement of COX-2 enzyme levels (1.6-fold change) (Figure 6A,B) and PGE_2_ release (2.8-fold change) into the basolateral medium (Figure 6C) was also observed. Pre-incubation with BFM and budesonide significantly reduced (*p* < 0.05) nuclear translocation of NF-κB p65 (65.6% and 29.9%, respectively) and restored its cytosolic levels (50.9% and 20.3%), as well as decreased COX-2 protein levels (79.3% and 40.2%) and PGE_2_ production (50.8% and 51.7%), compared to LPS-induced stimulation. Overall, combined treatment of BFM with budesonide exhibited effects comparable to those achieved with BFM incubation alone.

### 3.7. Pharmacological Interaction Between BFM and Budesonide

**Figure 7:**
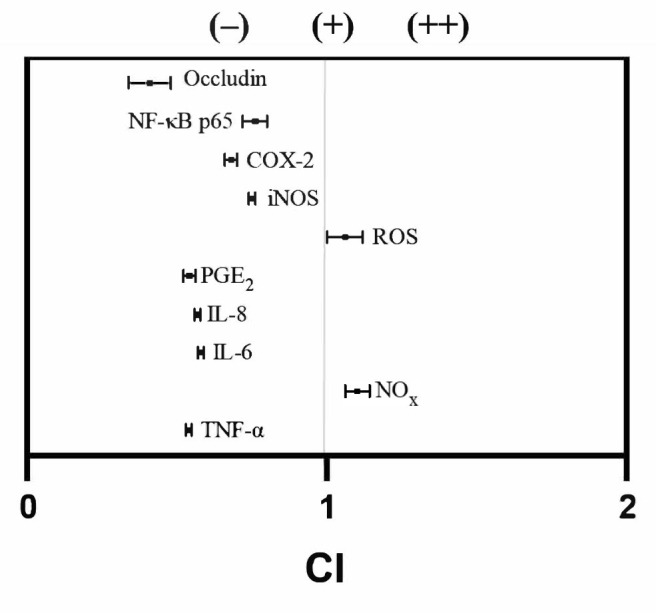
**Evaluation of pharmacological interactions between budesonide and BFM using CI analysis on different inflammatory markers.** Measurements were performed in the apical medium (differentiated Caco-2 cells: IL-8, NOx; RAW264.7 macrophages: IL-6, TNF-α, PGE_2_) and intracellularly (differentiated Caco-2 cells: iNOS, ROS, occludin; RAW264.7 macrophages: NF-κB p65, COX-2). A CI ratio less than 1 indicates antagonism (–), equal to 1 represents additivity (+), and greater than 1 demonstrates synergy (++). Data are expressed as mean ± SD (*n* = 3).

Calculation of the CI in in vitro assays is essential for understanding drug interactions—whether antagonistic, additive, or synergistic—in the treatment of IBD [43]. As described above, this approach suggests that the bioactive natural compounds in BFM exert anti-inflammatory effects in this in vitro model, showing responses comparable to those observed with budesonide. These findings indicate that BFM may represent a promising source of bioactive compounds with potential anti-inflammatory properties. Pharmacological interaction assessment between budesonide and BFM showed an antagonistic effect on most of pro-inflammatory markers, with CI values ranging from 0.41 ± 0.07 to 0.76 ± 0.04 (Figure 7). Nevertheless, an additive interaction was observed in the reduction of intracellular ROS production (CI, 1.06 ± 0.06), while a synergistic effect was demonstrated in the decrease of NOx release (CI, 1.10 ± 0.04).

## 4. Discussion

IBD represents a group of disorders characterized by excessive inflammatory responses in the gastrointestinal tract, with CD and UC being the two primary clinical manifestations [1]. The etiology of IBD is multifactorial, involving a complex interplay among genetic predisposition, environmental triggers, immune system dysregulation, and alterations in the composition and function of the intestinal microbiota [49]. Oxidative and nitrosative stress are key contributors to IBD pathogenesis. These processes are marked by the excessive production of reactive oxygen and nitrogen species (ROS and RNS), which contribute to tissue damage and the amplification of inflammation [2]. In IBD, a dysregulated balance between ROS overproduction and the body’s antioxidant defense mechanisms results in persistent oxidative stress and subsequent tissue injury [50,51,52]. By disrupting cellular homeostasis, oxidative stress activates multiple pro-inflammatory signaling pathways, further intensifying the disease process. Among these, a prominent pathway involves the nuclear translocation of NF-κB, a key transcription factor that regulates the expression of various pro-inflammatory mediators, including cytokines such as TNF-α, IL-8, IL-6, and IL-1β [53,54]. Additionally, the upregulation of pro-inflammatory enzymes such as COX-2 and iNOS exacerbates inflammation by increasing the local production of inflammatory mediators—including PGE_2_, NOx, and ROS—which significantly contribute to mucosal injury and the overall pathophysiology of IBD. A hallmark of IBD pathogenesis is the disruption of gut barrier integrity [55]. The intestinal epithelial barrier plays a crucial role in regulating permeability, preventing the entry of harmful agents while allowing nutrient absorption [56]. TJ proteins—such as occludin, claudins, and ZO-1—are essential for maintaining the structural and functional integrity of this barrier [57]. These proteins mediate the selective passage of molecules and prevent the translocation of harmful substances, a process dynamically regulated by intracellular signaling pathways and external modulators that influence TJ complexes [58]. Impairment of these regulatory mechanisms compromises barrier integrity, thereby contributing to the development and persistence of pathological conditions. Furthermore, occludin is a key target of redox-mediated processes [59]. Various biological models are employed to study intestinal inflammation, including in vitro, ex vivo, and in vivo approaches. Among these, in vitro models—particularly cell culture systems—are widely preferred for investigating mechanisms underlying intestinal inflammation due to their simplicity, reproducibility, and physiological relevance, especially when using human cell lines or co-culture systems [60,61]. As previously discussed, the increasing prevalence of IBD [62] underscores the need to identify novel alternatives to conventional treatments, including bioactive dietary compounds with anti-inflammatory potential. A recent in vitro investigation demonstrated that a HME obtained from Sicilian *Fraxinus angustifolia* Vahl exhibited significant antioxidant and anti-inflammatory properties. Specifically, the extract effectively attenuated ROS generation, restored intracellular GSH homeostasis, and suppressed the secretion of IL-8 and IL-6 in IL-1β-stimulated, differentiated Caco-2 cells. Comparison of the current study with previously reported analyses of *Fraxinus angustifolia* [11] manna shows notable differences in the concentration of individual phenolic compounds. While tyrosol and HT were detected in both studies, their absolute concentrations were lower in the current extract (16.66 ± 0.25 mg/kg and 10.33 ± 0.40 mg/kg, respectively) compared to the previous report (36.66 ± 0.25 mg/kg and 13.33 ± 0.40 mg/kg, respectively). Other compounds, such as catechin, luteolin 3,7-glucoside, and verbascoside, were also present at lower concentrations, whereas gallic acid, procyanidin B1, and elenolic acid were not detected in the current extract. These differences likely reflect natural variability among Manna sources, harvesting conditions, and extraction procedures. Importantly, our study extends these observations by analyzing the bioaccessible fraction after in vitro gastrointestinal digestion, allowing us to identify which phenolic compounds remain stable and potentially available for intestinal absorption. This provides a more biologically relevant assessment of the Manna phenolic profile and its nutraceutical potential, suggesting that manna may represent a promising source of bioactive compounds with potential anti-inflammatory properties. Moreover, among the phenolic constituents identified in the manna extract, HT, tyrosol, and secoiridoid derivatives—compounds also abundant in olive oil—were particularly notable, reflecting the chemotaxonomic relationship between the genera *Fraxinus* and *Olea* within the Oleaceae family [63]. Additionally, an ex vivo experiment demonstrated that olive oil-derived phenolics, once absorbed following consumption, prevent oxysterol-induced pro-inflammatory cytokine release and ROS formation in PBMCs by modulating the p38 and JNK signaling pathways [64]. These active components contribute to improving redox homeostasis and modulating pro-inflammatory cytokines by inhibiting the MAPK/NF-κB signaling axis [65]. Moreover, fraxetin, a hydroxycoumarin derived from the bark of *Fraxinus rhynchophylla*, has exhibited anti-proliferative effects by regulating the JAK2/STAT3 pathway, inducing S-phase cell cycle arrest, and triggering intrinsic apoptosis in colorectal adenocarcinoma cells [66]. In addition to these effects, its anti-inflammatory properties have been demonstrated in studies on *Fraxinus chinensis* stem bark, where fraxetin and other bioactive compounds suppressed NO production and reduced TNF-α and IL-6 secretion by inhibiting MAPK phosphorylation and IκBα degradation in LPS-stimulated RAW264.7 cells [67]. Building on this knowledge, our study utilized a co-culture model consisting of Caco-2 intestinal epithelial cells and RAW264.7 macrophages. Over the years, several studies have employed co-culture systems to assess the activity of various extracts and bioactive compounds, including those derived from BF obtained through in vitro gastrointestinal digestion [24,25,26,27,28,29,30,31,32,33,34,35]. In this investigation, we evaluated the anti-inflammatory properties of BFM, obtained through INFOGEST 2.0 gastrointestinal digestion of purified manna, which was processed using a patented method developed in our laboratory to preserve its bioactive components. This approach allows the evaluation of BFM’s anti-inflammatory effects while accounting for the chemical and enzymatic changes that manna undergoes during gastrointestinal digestion. Our results demonstrated that a dilution of BFM at 1/20 (*v*/*v*), equivalent to 6 mg manna/mL, which did not affect cell viability, was effective following a 90-min pre-treatment in differentiated Caco-2 cells. Based on an estimated intestinal volume of 640 mL [68], the amount of manna required to achieve this anti-inflammatory response corresponds to approximately 3.8 g/day. Supporting our findings, a recent study using a Caco-2/RAW 264.7 co-culture model demonstrated that an extract from acai berries enhanced the integrity of the tight junction barrier and mitigated intestinal inflammation by reducing the production of pro-inflammatory mediators such as IL-6, IL-8, and PGE_2_ [32]. Similarly, additional research using the same co-culture system showed that cinnamon subcritical water extract attenuated intestinal inflammation by downregulating the protein and expression levels of nitrite, PGE_2_, IL-6, IL-8, TNF-α, and NF-κB activity, while enhancing the assembly of TJ-related proteins [30]. In line with these studies, our findings demonstrate that pre-treatment with BFM significantly reduced IL-8 secretion (70.8%) in differentiated Caco-2 cells, as well as the release of IL-6 (43.1%) and TNF-α (83.1%) from RAW264.7 macrophages. Given the pivotal role of oxidative and nitrosative stress in IBD pathogenesis, BFM effectively reduced iNOS protein levels (48.2%), its downstream product NOx (33.2%), and intracellular ROS generation (26.4%) in Caco-2 cells. Considering that disruption of intestinal barrier integrity is a hallmark of IBD and that occludin plays a key role in maintaining TJ structure, our study focused on the expression of this protein. Substantial evidence suggests that occludin, a critical TJ component, is regulated by redox processes, including structural modifications, phosphorylation, and proteolytic degradation [59]. In this context, our results indicated that BFM restored occludin protein levels (18.3%), thereby contributing to improved epithelial integrity in the Caco-2 cell monolayer. We acknowledge that additional assays, such as paracellular flux studies with FITC-dextran or radiolabeled mannitol, as well as the evaluation of other tight junction markers (ZO-1, claudin-1), would further strengthen the assessment of barrier integrity. In the present study, we relied on TEER monitoring and occludin expression, and we explicitly recognize the absence of these additional measurements as a limitation. Furthermore, we examined the NF-κB–COX-2–PGE_2_ signaling cascade in RAW264.7 macrophages—a critical pathway in inflammation regulation—by assessing NF-κB activation through nuclear translocation of its p65 subunit. We also measured COX-2 protein levels, a key downstream effector of NF-κB, and quantified its enzymatic product, PGE_2_, a bioactive lipid mediator central to the inflammatory response. A recent study reported that the bioaccessible fraction (BF) of a plant sterol food supplement inhibited the NF-κB–COX-2–PGE_2_ axis in a Caco-2/RAW264.7 co-culture model [34]. Similarly, BFM reduced NF-κB p65 nuclear translocation (65.6%), COX-2 protein levels (79.3%), and PGE_2_ secretion (50.8%). These results suggest that manna may serve as a potential source of bioactive compounds with anti-inflammatory properties by targeting the NF-κB–COX-2–PGE_2_ signaling axis. Based on our data, the effective daily dose of 3.8 g required to achieve anti-inflammatory effects is substantially lower than the typical laxative dose of 30 g/day. Moreover, we compared the efficacy of manna with that of budesonide, a potent anti-inflammatory drug [69]. Unlike other corticosteroids, budesonide exhibits limited systemic effects due to extensive first-pass hepatic metabolism, which minimizes the risk of side effects. Clinically, budesonide is effective in treating mild to moderate CD [36]. Although BFM alone displayed strong anti-inflammatory and antioxidant activities comparable to those of budesonide, co-treatment revealed a range of interactions—antagonistic, additive, and synergistic. Budesonide exerts its anti-inflammatory effects mainly through glucocorticoid receptor-mediated transcriptional regulation, whereas BFM likely acts through antioxidant and redox-modulating pathways [70,71]. Such differences may result in partial interference at the level of downstream inflammatory signaling when both agents are co-administered, leading to reduced efficacy on certain mediators. Nevertheless, this interaction does not affect the intrinsic anti-inflammatory activity of BFM when used alone. Importantly, the combined treatment showed additive (ROS) and synergistic (NOx) effects, suggesting that the interaction is not uniformly antagonistic but rather pathway-dependent and context-specific. These findings suggest that manna may represent a promising source of bioactive compounds with potential anti-inflammatory properties, particularly in the early stages of the disease, when pharmacological intervention may not yet be necessary.

This study presents some limitations that should be acknowledged to properly contextualize the findings. While the anti-inflammatory effects of BFM were clearly demonstrated through modulation of the NF-κB–COX-2–PGE_2_ axis, a more comprehensive mechanistic exploration—including upstream signaling events and downstream molecular targets—was not conducted and could provide further insights into the pathways involved. Determination of TPC and phenol profile of the digested fraction revealed a quantitative reduction in polyphenols. However, this decrease does not necessarily imply a loss of overall biological activity, as structural changes during digestion may enhance certain bioactive properties despite the reduction in total polyphenols. Similar findings have been reported in digested grape and wine, where, although polyphenol content decreased significantly after in vitro digestion, the antioxidant bioactivity measured at the cellular level (H_2_O_2_-stimulated Caco-2 cells) was maintained, indicating that compositional shifts do not abolish functional effects [72]. Although the phenolic profile of BFM was characterized, these compounds alone are unlikely to fully account for the observed biological effects. BFM represents a complex mixture generated after in vitro gastrointestinal digestion and may contain additional bioactive constituents, including D-mannitol, D-mannose, and other low-molecular-weight carbohydrates previously associated with anti-inflammatory activity [17]. Moreover, the presence of higher-molecular-weight compounds, such as polysaccharides and polysaccharide–protein complexes, cannot be excluded and may contribute to the overall biological activity. However, these macromolecular fractions were not specifically quantified in the present study, which represents an additional limitation. Additionally, the bioavailability and metabolic fate of the bioactive compounds, particularly in relation to phase II metabolism (e.g., glucuronidation and sulfation), remain unaddressed, thereby restricting the extrapolation of the in vitro findings to in vivo conditions. Another important aspect not captured by the current model is the potential interaction between BFM and the gut microbiota, a key player in IBD pathogenesis. In particular, components of manna such as mannitol have been reported to exert prebiotic effects [73], which could positively modulate the microbiota and, consequently, impact IBD progression. However, these effects could not be assessed in our cellular model, which lacks microbiota. Future studies incorporating microbiome-focused analyses would be valuable to explore how BFM may influence microbial composition or function. Overall, although these limitations do not diminish the relevance of the current results, they highlight areas for future research that could strengthen the mechanistic understanding and translational potential of BFM as a dietary strategy for managing intestinal inflammation. In particular, further in vivo and clinical studies are required to confirm the biological relevance and potential applications of Manna.

## 5. Conclusions

Research in the field of IBD continues to focus on elucidating the mechanisms underlying intestinal inflammation and developing innovative therapeutic strategies. Given the limited efficacy and adverse effects often associated with conventional treatments, there is growing interest in natural products as alternative or complementary approaches. Phytochemicals, particularly those abundant in plant-based foods characteristic of the Mediterranean diet, have shown the capacity to modulate the intestinal inflammatory response. Although manna has been used in traditional medicine for centuries, this study is the first to experimentally demonstrate the anti-inflammatory activity of BFM in an in vitro model of intestinal inflammation. The findings suggest that manna may act as a functional food capable of preventing or attenuating intestinal inflammation by downregulating the NF-κB–COX-2–PGE_2_ signaling pathway. This nutraceutical potential, supported by the presence of bioactive compounds and the absence of known contraindications, highlights manna as a promising candidate for the development of targeted dietary interventions in IBD management. Overall, the results provide a solid foundation for expanding scientific knowledge on manna and its health-promoting properties. However, further in vivo studies are necessary to validate these findings and evaluate their translational relevance in the context of human health.

## Figures and Tables

**Table 1:** HPLC-DAD analysis of main phenolic compounds in Manna before (undigested) and after (BFM) in vitro gastrointestinal digestion.

Compound	Rt (min)	Undigested Manna (mg/kg)	BFM (mg/kg)	Bioaccessibility (%)
Hydroxytyrosol	9.005	10.33 ± 0.40 ^aA^	5.01 ± 0.36 ^aB^	48.5 ± 0.38 ^a^
Tyrosol	12.677	16.66 ± 0.25 ^bA^	6.01 ± 0.29 ^aB^	36.1 ± 0.26 ^b^
Catechin	16.361	3.86 ± 0.25 ^cA^	1.23 ± 0.08 ^bB^	31.9 ± 0.13 ^c^
Fraxetin	22.631	<LOQ	<LOQ	Not calculated
Luteolin 3,7-glucoside	31.571	0.50 ± 0.05 ^dA^	0.18 ± 0.01 ^bA^	36.0 ± 1.33 ^b^
Verbascoside	38.052	2.50 ± 0.22 ^cA^	1.04 ± 0.05 ^bA^	41.6 ± 1.26 ^d^
Total phenolics	Not applicable	33.85 ± 1.09 ^A^	13.47 ± 0.68 ^B^	39.8 ± 0.88

BFM: bioaccessible fraction of manna; LOQ: limit of quantification. Different lowercase letters (a–d) within the same column indicate statistically significant differences among phenolic compounds, while different uppercase letters (A,B) within the same row indicate statistically significant differences between undigested and digested samples (*p* < 0.05). For fraxetin, “Not calculated” indicates that bioaccessibility could not be determined due to both undigested and digested values were below the LOQ. For total phenolics, “Not applicable” indicates that retention time is not defined, as this value does not correspond to a single chromatographic compound.

**Table 2:** Evaluation of the effects of BFM and BD on viability of 8-day differentiated Caco-2 cells.

Cell Viability (% vs. Control)			
Dilution (*v*/*v*)	30 min	60 min	90 min
**Control cells**	100 ± 4.6	100 ± 4.0	100 ± 4.4
**BD**			
1/10	99.5 ± 1.4	97.9 ± 8.1	91.6 ± 2.8
1/20	103.7 ± 0.8	94.7 ± 2.5	98.6 ± 1.2
1/30	105.6 ± 1.2	96.8 ± 3.9	97.1 ± 2.0
**BFM**			
1/10	73.9 ± 6.1 *	73.7 ± 0.7 *	70.7 ± 3.1 *
1/20	98.3 ± 1.4	97.7 ± 1.8	96.1 ± 0.8
1/30	98.4 ± 1.2	97.8 ± 0.5	96.4 ± 2.0

Experiments were conducted using different dilutions (1/10, 1/20, and 1/30, *v*/*v*) and exposure times (30, 60, and 90 min). Data are expressed as mean ± SD of three experiments conducted in triplicate. * Indicates statistically significant differences (*p* < 0.05) compared to control (untreated) cells, (*T*-test).

## Data Availability

The original contributions presented in this study are included in the article/Supplementary Materials. Further inquiries can be directed to the corresponding authors.

## Supplementary Materials

The following supporting information can be downloaded at: https://www.mdpi.com/article/10.3390/antiox15050601/s1, Figure S1: Superimposed HPLC-DAD chromatograms recorded at 280 nm under identical analytical conditions for: digested Manna (blue), undigested Manna extract (green), and digestion blank (yellow); Figure S2: Full-length Western blot membranes corresponding to the representative original experiments.

## Abbreviations

The following abbreviations are used in this manuscript:

BD	Blank of digestion
BFM	Bioaccessible fraction of manna
BSA	Bovine serum albumin
DCFDA	2′,7′-dichlorofluorescein diacetate
DMEM/HG	Dulbecco’s Modified Eagle’s Medium High Glucose
DMSO	Dimethyl sulfoxide
DSS	Dextran sodium sulfate
DTT	Dithiothreitol
ELISA	Enzyme-Linked ImmunoSorbent Assay
GSH	Glutathione
HRP	Horseradish peroxidase
HT	Hydroxytyrosol
IB	Immunoblot
IκBα	Inhibitor of nuclear factor kappa B
JNK	Jun N-terminal kinase
MAPK	Mitogen-activated protein kinase
MTT	3-(4,5-dimethylthiazol-2-yl)-2,5-diphenyl-tetrazolium bromide
NED	N-(1-naphthyl)ethylenediamine
NO	Nitric oxide
PBMCs	Human peripheral blood mononuclear cells
PVDF	Polyvinylidene difluoride
SDS-PAGE	Sodium dodecyl sulfate polyacrylamide gel electrophoresis
TBS	Tris-buffered saline
TBST	TBS containing Tween
TJ	Tight junctions
TNF-α	Tumor necrosis factor α
ZO-1	Zonula occludens-1
