# Supplementary material for: Anti-Inflammatory Activity of In Vitro Digested Manna in a Caco-2 and RAW264.7 Cells Co-Culture Model of Inflammatory Bowel Disease
Source: Antioxidants (Basel). 2026 May 9;15(5):601. doi: 10.3390/antiox15050601 (PMC13203731; doi:10.3390/antiox15050601)
Supplement: Supplementary file 1 [file antioxidants-15-00601-s001.zip › antioxidants-4281700-supplementary.pdf]

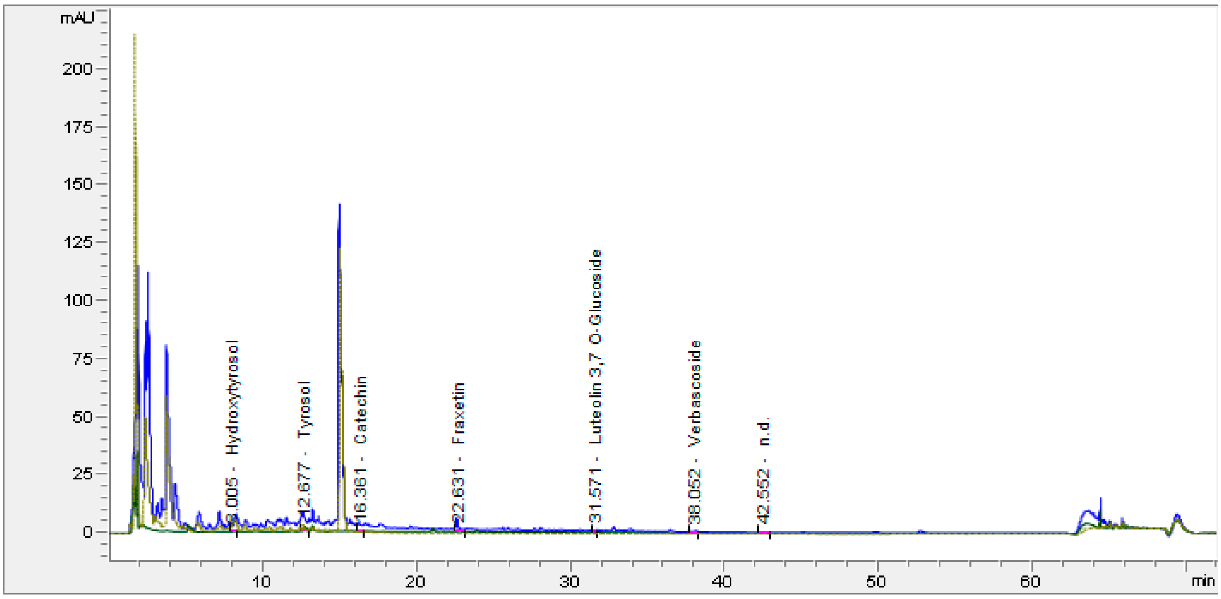

Figure S1. Superimposed HPLC-DAD chromatograms recorded at 280 nm under identical analytical conditions for: digested Manna (blue), undigested Manna extract (green), and digestion blank (yellow). The chromatogram illustrates the presence of phenolic compounds (hydroxytyrosol, tyrosol, catechin, fraxetin, luteolin 3,7-glucoside, and verbascoside) in both the undigested extract and the bioaccessible fraction, while no corresponding peaks are detected in the digestion blank. Retention times of major compounds are indicated.

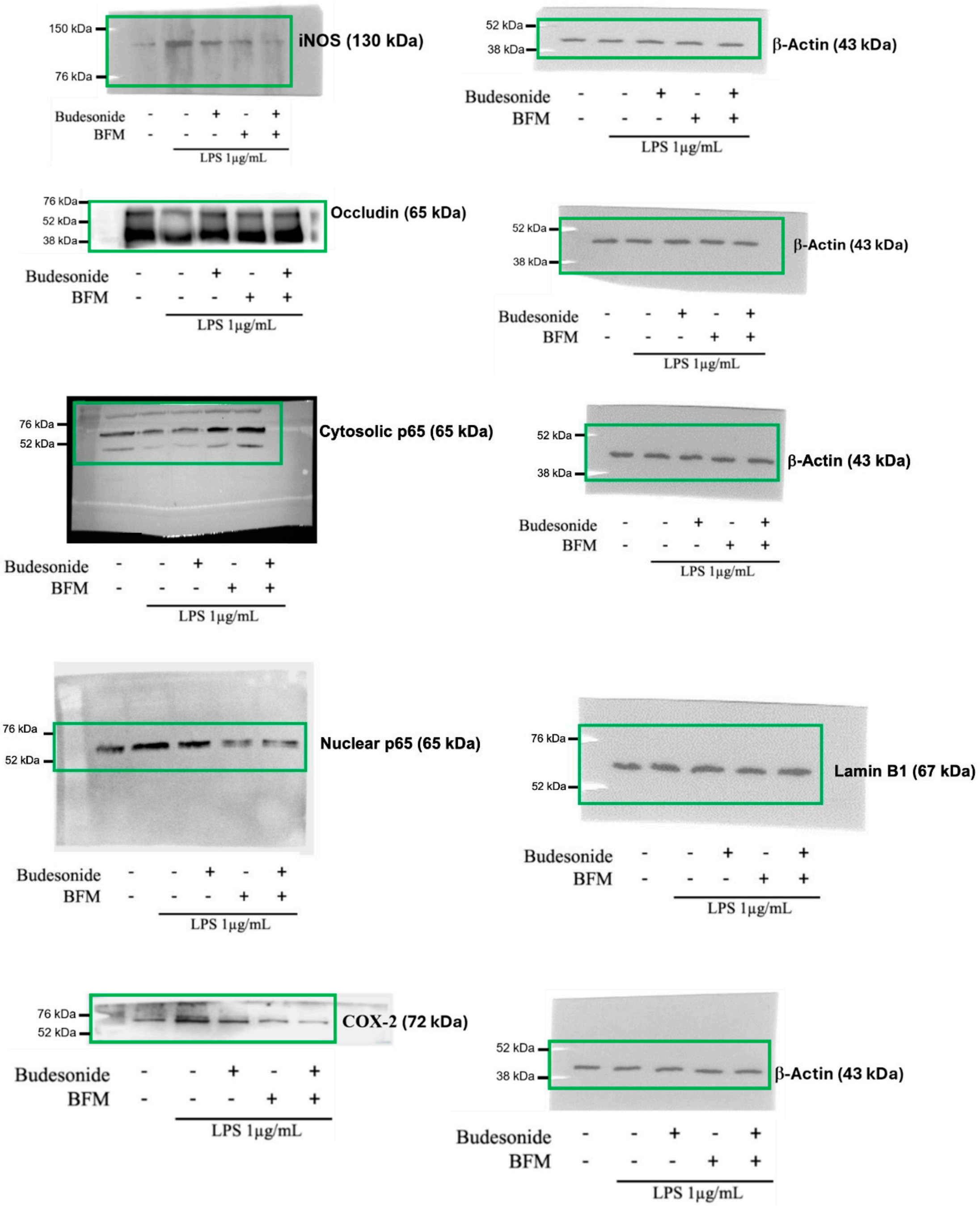

Pre-stained molecular weight marker that provides visible reference bands on the membrane by eye, prior to immunodetection.

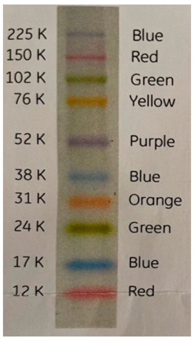

Figure S2. Full-length Western blot membranes corresponding to the representative original experiments. All samples and corresponding controls were run under identical experimental conditions. No lane rearrangement was performed. Exposure conditions were optimized to avoid signal saturation.
